# Neuroprotection, Recovery of Function and Endogenous Neurogenesis in Traumatic Spinal Cord Injury Following Transplantation of Activated Adipose Tissue

**DOI:** 10.3390/cells8040329

**Published:** 2019-04-08

**Authors:** Stephana Carelli, Toniella Giallongo, Federica Rey, Mattia Colli, Delfina Tosi, Gaetano Bulfamante, Anna Maria Di Giulio, Alfredo Gorio

**Affiliations:** 1 Laboratory of Pharmacology, Department of Health Sciences, University of Milan, Via A. di Rudinì 8, 20142 Milan, Italy; toniella.giallongo@unimi.it (T.G.); federica.rey@unimi.it (F.R.); mattiacolli@live.it (M.C.); annamaria.digiulio@unimi.it (A.M.D.G.); 2 Pediatric Clinical Research Center “Fondazione Romeo e Enrica Invernizzi”, University of Milan, via Giovanni Battista Grassi 74, 20142 Milan, Italy; 3 Pathological Anatomy, Department of Health Science, University of Milano, Via A. di Rudini’ 8, 20142 Milano, Italy; delfina.tosi@unimi.it (D.T.); gaetano.bulfamante@unimi.it (G.B.)

**Keywords:** spinal cord injury, adipose tissue, mechanical activation, cell therapies, inflammation, neuroprotection, neurogenesis

## Abstract

Spinal cord injury (SCI) is a devastating disease, which leads to paralysis and is associated to substantially high costs for the individual and society. At present, no effective therapies are available. Here, the use of mechanically-activated lipoaspirate adipose tissue (MALS) in a murine experimental model of SCI is presented. Our results show that, following acute intraspinal MALS transplantation, there is an engraftment at injury site with the acute powerful inhibition of the posttraumatic inflammatory response, followed by a significant progressive improvement in recovery of function. This is accompanied by spinal cord tissue preservation at the lesion site with the promotion of endogenous neurogenesis as indicated by the significant increase of Nestin-positive cells in perilesional areas. Cells originated from MALS infiltrate profoundly the recipient cord, while the extra-dural fat transplant is gradually impoverished in stromal cells. Altogether, these novel results suggest the potential of MALS application in the promotion of recovery in SCI.

## 1. Introduction

Spinal cord injury (SCI) is a devastating disease mainly caused by high-energy trauma. Patients with SCI always suffer from paralysis, locomotor and sensory dysfunction, urinary incontinence, or gastrointestinal dysfunction [1]. The impairment and disability derived from SCI leads to enormous personal suffering and substantial costs to society [2,3,4], with an incidence of 27–83 per million in the US and 10–30 per million in Europe [5,6]. In addition to the acute traumatic mechanical injury, there is the activation of secondary neurodegenerative mechanisms leading to the final damage of the cord [4,7,8,9]. There is currently no curative therapy, and in the acute phase, the intervention is often limited to surgical stabilization and decompression with possible high-dose corticosteroid treatment [3,10]. The use of acute erythropoietin was suggested, and recent small studies are suggestive of possible positive outcomes [11,12,13]. Many different approaches had been developed with unfortunately poor clinical outcomes [14], and even cellular and embryonic tissue transplantation were suggested and studied for a long time with the aim to improve recovery of function through the partial replacement of neural tissue loss or the creation of a favorable environment for survival to injury and regrowth [13,14,15,16,17,18,19]. Up to now, no favorable clinical results are known and long-term safety remains a significantly open unmet medical need [16,17,18,20,21]. Curtis and coworkers [18] recently conducted a one-year long phase I trial in six patients with subacute spinal cord injury. This work showed that one year post-transplantation, there were no surgical, medical, or neurological complications in treated patients, thus indicating that the timing or procedure for the cell transplantation was safe. Until now, no positive clinical outcomes could be observed and this is a highly limiting step for the suggested therapy. Adipose tissue is a source of adult stem cells that show neuronal markers [22,23]. The advantages of using subcutaneous adipose tissue in comparison to other mesenchymal stem cell (MSC) sources have recently been investigated and confirmed. Indeed, this tissue can easily be accessed with low patient discomfort and the isolation of stem cells from it requires simpler and more efficient procedures [24]. It was suggested that implantation of adipose tissue-derived cells promoted functional recovery and revascularization in experimental spinal cord injury [25,26]. We recently demonstrated that the application of mechanical forces to the adipose tissue abolishes the inflammatory properties of fat and favors the expression of anti-inflammatory cytokines, such as TNF-alpha stimulated gene-6 (TSG-6) [23], which has been shown to exert protective effects in disease experimental models [27]. The mechanical activation of the adipose tissue could promote post-translational pathways implicated in mechano-transduction [28]. Indeed, stretch-activated ion channels, growth factors, cytoskeleton filaments, focal adhesions, and tyrosine kinases, could all be involved in the translation of signals from mechanical forces into biochemical events, with downstream signals communicating key messages to the nucleus and subsequently altering gene expression [23,29,30,31,32,33]. 

In the present study, we thus aimed to investigate the regenerative potential of the mechanically activated lipoaspirate adipose tissue (MALS) transplanted into the lesion epicenter in a murine experimental model of SCI, immediately after the contusion [13,15,34]. We show that MALS results engrafted two weeks after transplantation and promotes recovery of motor function, which is already evident at 24 hours after the insertion in the lesion site. The positive effects on functional recovery increases during the following days. This is associated with strong improvements in tissue preservation and counteraction of the local inflammatory response into the damaged spinal cord. We observe an increased number of cells positive to neuronal markers at the lesion site, where MALS has infiltrated. We hypothesize that the MALS convey for a sustained delivery of therapeutic agents that counteracts the acute local inflammatory response following spinal cord injury. We, thus, postulate that this allows a more delayed effect due to the synergic differentiation mechanism of ependymal stem cells.

## 2. Materials and Methods

### 2.1. Animal Care

In this study, adult CD1 male mice 25–30 g in weight (Charles River, Calco, Italy) were used. The animals were kept in standard conditions (22 ± 2 °C, 65% humidity, and artificial light between 8:00 a.m. and 8:00 p.m.) for at least three days before the experiments. All of the procedures were approved by the Review Committee of the University of Milan and met the Italian Guidelines for Laboratory Animals that gave its approval to the study (no. 3/2013), which conform to the European Communities Directive (2010/63/UE).

### 2.2. Tissue Mechanical Activation

Voluntary patients undergoing procedures of elective liposuctions were anesthetized locally with lidocaine (AstraZeneca, London, UK). The procedure requires an infiltration step, with the infusion of a solution of saline and epinephrine (2 μg/mL) (Key Customer Solutions S.A.S, Basiglio, Milan, Italy) into the adipose compartment. This leads to a minimization of blood loss and the reduction of infiltration by peripheral blood cells. From this procedure, it is possible to obtain a particular kind of adipose tissue named lipoaspirate adipose tissue (lipoaspirate, LS) [23]. The activation of lipoaspirate has been performed as described in our previous paper [23]. Briefly, 10–15 mL of lipoaspirated adipose tissue was activated by orbital shaking (97× *g*) for 10 min at room temperature. The product has been termed mechanically-activated lipoaspirate (MALS; WO 2017/115289 Al). Elective liposuction procedures were performed in voluntary patients with full knowledge of the events. An informed consent was supplied and signed by each patient. 

### 2.3. Spinal Cord Injury, Setting of Experimental Animal Groups

Traumatic SCI was performed in CD1 mice at the T8 level using a commercially available Infinite Horizon Impactor device (Precision Systems and Instrumentation, LLC, Lexington, KY, USA), as previously described and surgery on the animals was performed with minor modifications [34]. Briefly, animals were anesthetized for five minutes before the surgery with 2.5% isoflurane in oxygen (1 L/min; Farmagricola, San Donato, Milan, Italy). A dorsal vertical incision was made through the skin from T7 to T12 and the superficial fat pad was removed. The T7 and T10 bilateral paravertebral muscles were cut and a laminectomy was performed, exposing the spinal cord. The impactor tip was then positioned just above the cord and a force of 70 Kdyne was applied for 1 s to the spinal cord. After the contusion, LS or MALS tissue were immediately administered at the lesion site. Then, the muscles and the skin were sutured as previously reported [34]. Experimental animals groups were three: (1) lesioned mice (LES; n = 16), (2) lesioned mice transplanted with lipoaspirate (LS; n = 16) and (3) lesioned mice transplanted with mechanically-activated lipoaspirate (MALS; n = 16; [23]).

### 2.4. RNA Extraction and Real Time-PCR

Thirty-five days after transplant animals were sacrificed and cords were dissected for subsequence gene expression analysis (n = 6 mice for each group). Tissue homogenates were derived from five regions: the lesion epicenter and 1 cm and 2 mm both rostral and caudal to the lesion site [13,15]. The distance from the lesion was evaluated using a square ruler in order to always have a reproducible distance from the lesion epicenter and an analogous thickness of the tissue homogenate. Specifically, each biopsy was 1mm thick, starting to cut 0.5 mm below and finishing 0.5 mm above the desired site (e.g., setting 0 as the lesion epicenter, the central section was obtained sectioning −0.5 mm and +0.5 mm from the lesion). The different areas were dissected out rapidly, frozen on dry ice, and stored at −80 °C until further processing. Total RNA was extracted using TRIZOL® reagent (Life Technologies, Carlsbad, CA, USA) following manufacturer’s instructions, quantified and processed as previously described [35,36]. Total RNA (1 μg) was reverse transcribed using iScript cDNA synthesis kit (Bio-Rad, Segrate, MI, Italy) and Real-Time PCR was performed with StepOnePlus^™^ Real-Time PCR System (Thermo Fisher, Waltham, MA, USA) using iQ SYBR Green Supermix (Bio-Rad). Primers were designed using Oligo Perfect Designer Software (Life Technologies) and the sequences are reported in Table 1. Amplification conditions were as follows: AmpliTaq activation 95 °C for 10 min; PCR denaturation step 95 °C for 15 sec; PCR annealing and elongation step 60 °C for 1 min; 40 cycles of PCR were performed. Genes were quantified in triplicates, and 18S was used as housekeeping gene. Gene expression was calculated using the 2^−ΔCt^ method.

### 2.5. Behavioral Tests and Hind Limb Function

Functional recovery evaluations were assessed in a blinded fashion as described by Basso and co-workers [37]. Neurological function was evaluated first 24 h after injury and then twice a week for four weeks. Locomotor function and hind limb recovery after contusion were evaluated with the open field test according to the Basso mouse rating scale. The methods used are well known in the field of behavioral evaluation of recovery of function after SCI [37]. For behavioral experiments 10 animals for all experimental groups were used: contusion 70 Kdyne; contusion 70 Kdyne + lipoaspirate (LS); contusion 70 Kdyne + mechanically-activated lipoaspirate (MALS). 

### 2.6. Immunohistochemistry and Quantitative Analysis

Immunohistochemistry was carried as previously reported [38]. Briefly, tissues were formalin-fixed and paraffin-embedded. A cryostat (set at −28 °C) was used to cut serial coronal sections (18 µm) of the spinal cord (Leica, Buccinasco, MI, Italy) at −28 °C. Sections were rinsed twice in 0.1 mol/mL PBS and then incubated in 3 g/L hydrogen peroxide diluted in 0.1 mol/L PBS for 15 min. This was done in order to block the endogenous peroxidase activity. Sections were washed twice more in 0.1 mol/L PBS and then blocked in 1 g/L normal goat serum dissolved in 0.1 mol/L PBS for 1 h. The sections were then incubated with antibodies specific for nestin (1:200; Millipore, Burlington, MA, USA), beta-tubulin III (beta-tub III; 1:500; Covance, Princeton, NJ, USA), Map2 (1:200; Millipore), GFAP (1:1000; Covance), CD68 (1:200; Abcam, Cambridge, UK) in 0.1 mol/L PBS overnight at +4 °C. Sections were washed three times in 0.1 mol/L PBS, and then incubated for 1 h in anti-rabbit and anti-mouse antibody (1:200), diluted in 0.1 mol/L PBS. The antigen-antibody complex was revealed with the avidin-biotin complex method using as chromogen the 30,30-diaminobenzidine (Sigma Chemical. Co., St Louis, MO, USA) and 0.01 g/L hydrogen peroxide in 0.1 mol/L PBS. When the product turned dark brown the reaction was arrested by washing with PBS. Sections incubated without primary antibody were used as negative control and they did not show any detectable immunoreactivity. 

Immunofluorescence analysis was also performed on 20 μm coronal sections obtained as above using a cryostat set at −28 °C (Leica). Slides were rinsed with PBS and treated with blocking solution (10% NGS, 0.2% Triton X-100) as previously described [13,39]. The following primary antibodies were used: MBP (1:500; Millipore), DCX (1:200; SantaCruz, Dalls, TX, USA) Adiponectin (1:50; Abcam), Leptin (1:200; Abcam). The secondary antibodies used were: 546 goat anti-rabbit IgG (1:200; Alexa, Thermofisher, Waltham, MA, USA), 546 donkey anti-goat IgG (1:200; Alexa), 488 goat anti-rat IgG (1:200, Alexa), 488 donkey anti-rabbit IgG (1:200, Alexa). Images were acquired using standardized confocal microscopy (Leica SP2 confocal microscope with He/Kr and Ar lasers). The ImageJ software (NIH. Bethesda, MD, USA) was used for microphotographic digital analysis [13,39,40]. The positive pixels were quantified against versus the negative background eliciting an index score, which includes fibers and neuronal [13]. The microscope light intensity of the laser was the same for all sections analyzed and for determining the background optical density. For the quantification of positive cells, the number of cells positive to the staining was counted with respect to the total nuclei number. This was done by means of ImageJ software following an already-described protocol [41].

### 2.7. Fluoro-Ruby Protocol

Fluoro-Ruby is a fluorescent rhodamine-conjugated dextran, widely used in literature, and already previously used by our group, to study in vivo axonal transport within the central nervous system (CNS) [13,42]. The preparation of the Fluoro-Ruby (Millipore) solution was done as previously described [13]. Fluo-Ruby was administered with an intraspinal injection at the level of the dorsal funiculus rostral to the lesion epicentre, at T6/T7 using a 5 µL Hamilton microsyringe 25 days after treatment. Injection volumes ranged from 0.5–1 µL and the injections were gradually performed over a period of 10–15 minutes [13]. The animals were then allowed to recover and perfused 10 days after the tracer injection. The perfusion was done with neutral buffered formaldehyde as previously described. The spinal cord was then removed, post-fixed overnight in the same fixative solution, and then included in OCT (Killik Bio-Optica, Milano, Italy). The cords were then sectioned by means of a microtome (Zeiss, Oberkochen, Germany) cutting sections 18 µm in thickness. This analysis was performed on 3 animals per experimental group: 1) lesioned mice, 2) lesioned mice transplanted with LS, and 3) lesioned mice transplanted with MALS.

### 2.8. BrdU Labeling

BrdU was administered intraperitoneally at 3 μL/g body weight from a stock solution of 15 mg/ml BrdU (Sigma-Aldrich, St. Louis, MO, USA) following manufacturer’s instructions and previously reported methods [43]. The injection was performed 25 days after treatment and animals were perfused 10 days after BrdU injection with neutral buffered formaldehyde as previously reported. The spinal cords were then removed, post-fixed overnight in the same fixative solution and included in OCT (Killik Bio-Optica). The cords were then sectioned by means of a microtome (Zeiss) with thickness set at 18 µm. Paraformaldehyde-fixed sections were denatured in 4 N HCl (15 min), neutralized in 100 mM sodium tetraborate pH 9.0 (15 min) and stained with monoclonal BrdU antibody (1:100, Abcam) overnight at 4 °C. The next day they were washed with PBS three times for 5 min each, the slides were incubated with AlexaFluor 488 conjugated anti-rat IgG antibody (1:100, Jackson Immunoresearch, West Grove, PA, USA) for 2h at RT. The ImageJ software was used for microphotographic digital analysis [13,39,40]. The positive pixels were quantified against versus the negative background eliciting an index score, which includes fibers and neuronal [13]. The microscope light intensity of the laser was the same for all sections analyzed and for determining the background optical density. This analysis was performed on three animals per experimental group: (1) not treated lesioned mice (LES); (2) lesioned mice transplanted with LS; and (3) lesioned mice transplanted with MALS.

### 2.9. Statistical Analyses

Data were expressed as mean ± S.D. Multiple group’s comparison were made by one-way ANOVA followed by Bonferroni’s multiple comparisons test to assess statistical significance versus respective control. In prospective analysis (time) of animals’ recovery of function, data were compared with two-way ANOVA test. The analyses were performed using Prism 5 software (GraphPad Software Inc., La Jolla, CA, USA.) assuming a *p* value less than 0.05 as the limit of significance.

## 3. Results

### 3.1. Mechanically Activated Lipoaspirate Engraftment Shows Anti-Inflammatory Properties in Spinal Cord Injury

**Figure 1:**
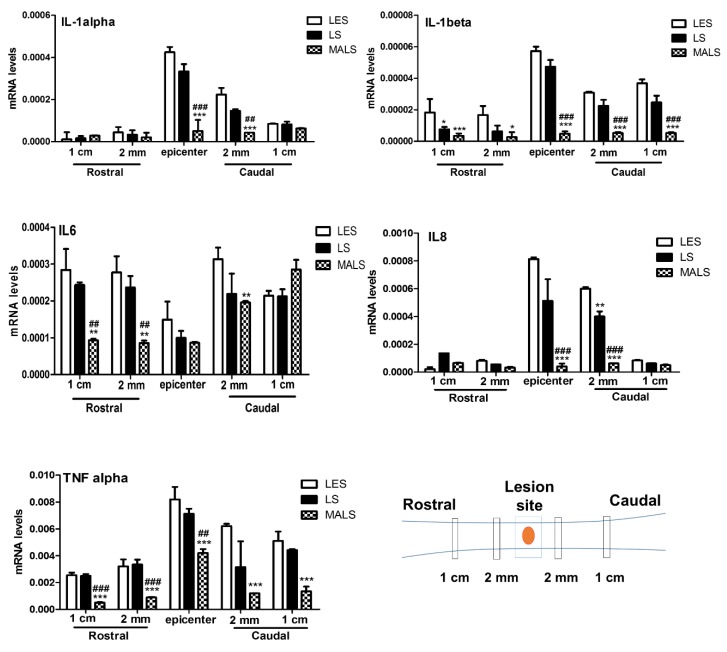
MALS tissue administration reduces the expression of pro-inflammatory cytokines. 35 days after transplantation, mRNA levels of IL-1 alpha, IL-1 beta, TNF-alpha, IL-6 and IL-8 were quantified at the lesion epicenter and 2 mm and 1 cm caudal and rostral to the lesion site of animals transplanted with Mechanically activated lipoaspirate (MALS), or with lipoaspirated tissue (LS) or in lesioned non- treated animals (LES). 18S was used as housekeeping gene. The evaluation was performed in triplicate on six different spinal cord samples for each condition. The data reported in the histogram are expressed as mean ± SD and statistical analysis was performed with Student’s t-test (n = 6). ***p < 0.001; ** *p* < 0.01; * *p* < 0.05 vs. LES. ### *p* < 0.001; ## *p* < 0.01 vs LS.

It is now established that the therapeutic effect of stem cells can consist in them exerting a potent effect of counteracting neuroinflammation [13,15,23,44]. We, thus, aimed at investigating the anti-inflammatory properties of Mechanically Activated Lipoaspirate (MALS) at the lesion site of Spinal Cord Injury (SCI). We assayed the expression of pro-inflammatory cytokines, which represent the major targets of pharmacological agents aimed at taming the inflammatory response in neurodegenerative disorders. The mRNA levels of IL-1 alpha, IL-1beta, TNF-alpha, IL6, and IL-8 were assayed by real time-PCR and their expression levels were compared between lesioned mice (LES), lesioned mice transplanted with lipoaspirate (LS), and lesioned mice transplanted with mechanically-activated lipoaspirate (MALS). Thirty-five days after contusion LES-, LS-, or MALS- transplanted animals were sacrificed (see Section 2 for details) and a small biopsy (1 mm) was micro-dissected from the following different five areas of the spinal cord: 1 cm and 2 mm rostral and caudal to the lesion site and the lesion epicenter (please see the schematic in Figure 1). In all investigated biopsies, the assayed chemokines resulted decreased in the cord of the lesioned animals treated with MALS respect to both the control untreated lesioned mice (LES) and LS treated mice. This effect is significantly evident in the epicenter and caudal area of the transplanted cord. In particular, IL-1 alpha was reduced in the epicenter and 2 mm caudal to the lesion site, whereas IL-1 beta and TNF-alpha were decreased in all areas analyzed. IL-6 was reduced 1cm and 2 mm rostral to the lesion site and 2 mm caudal to the lesion site. IL-8 was reduced in the epicenter of the lesion and 2 mm caudal to the lesion site (Figure 1). Concordantly with these data, a decrease of CD68 positive cells was observed at the lesion site in MALS treated mice, suggesting a decreased macrophage infiltration (Figure S1). Together, the results indicate that MALS protects the contused spinal cord by inhibiting the expression of pro-inflammatory cytokines.

### 3.2. Lipoaspirate and MALS Engraftment Improve Recovery of Hind Limb Function 

**Figure 2:**
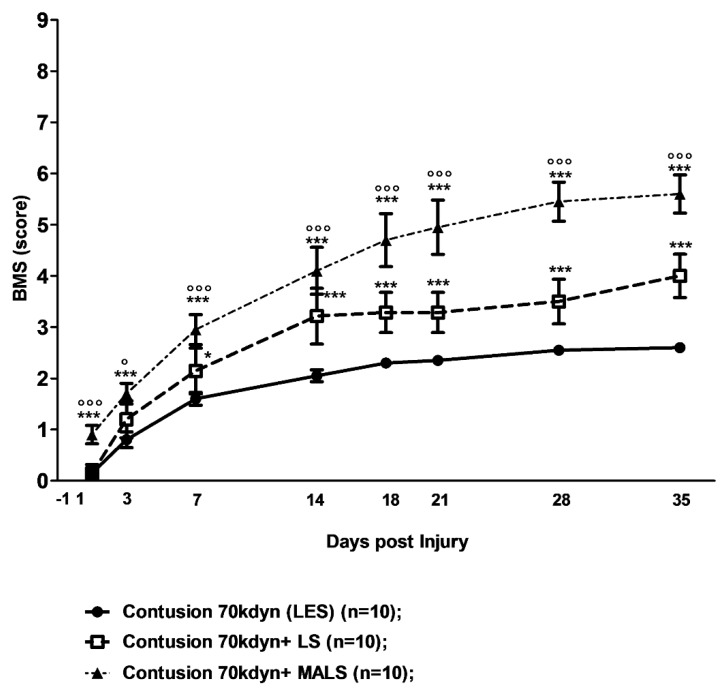
MALS promotes recovery of hind limb function. The evaluation of hind limb motor functional recovery was determined by the open field locomotion test [37]. Lesioned mice showed a remarkable and long-lasting improvement when treated with MALS. The groups were randomized, and the analysis was performed in double-blind fashion. Values represent mean ± SD (n = 10). Statistical differences were evaluated by means of two-way ANOVA test followed by Bonferroni’s post-test. *** *p* < 0.001; * *p* < 0.05 versus LES (lesioned untreated mice); °°° *p* < 0.001; ° *p* < 0.05 versus LS (lesioned mice treated with lipoaspirate).

The evaluation of hind limb function recovery performed by open field locomotion test, Basso Mouse Scale (BMS; Figure 2) [37]. All animals involved in the study, tested the day prior to the contusion, obtained the top score (nine points) in the BMS scale. The 70 Kdyne traumatic impact on the spinal cord caused a loss of ability in hind limb function of lesioned mice, which was followed by a progressive gradual recovery reaching its maximum in five weeks (2.60 ± 0.32 points of the BMS scale; n = 10) in complete accordance with previously published results [13,15] (Figure 2). This corresponds to plantar placing of the paw with or without weight support or occasional, frequent, or consistent dorsal stepping, but no plantar stepping [37]. When animals were transplanted with lipoaspirate adipose tissue (LS), their recovery was significantly better than the LES group already at seven days, and reach a BMS score of up to 3.6 ± 1.1 points on the BMS scale at day 35 after transplantation (n = 10). This corresponds to occasional plantar stepping. After MALS engraftment, the functional recovery improved even more, starting to be significant already the day after transplantation (Figure 2). The behavioral improvement was particularly evident between days 7 and 14 after SCI, and ameliorated again from 14 days after SCI until the end of the observational period (35 days). At this time point, the BMS score significantly improved for the MALS transplanted animals up to 5.70 ± 0.37 points, which corresponds to frequent or consistent plantar stepping, some coordination, and paws parallel at initial contact (Figure 2 and Table S1). 

### 3.3. MALS Engraftment Promotes White Matter Sparing 

**Figure 3:**
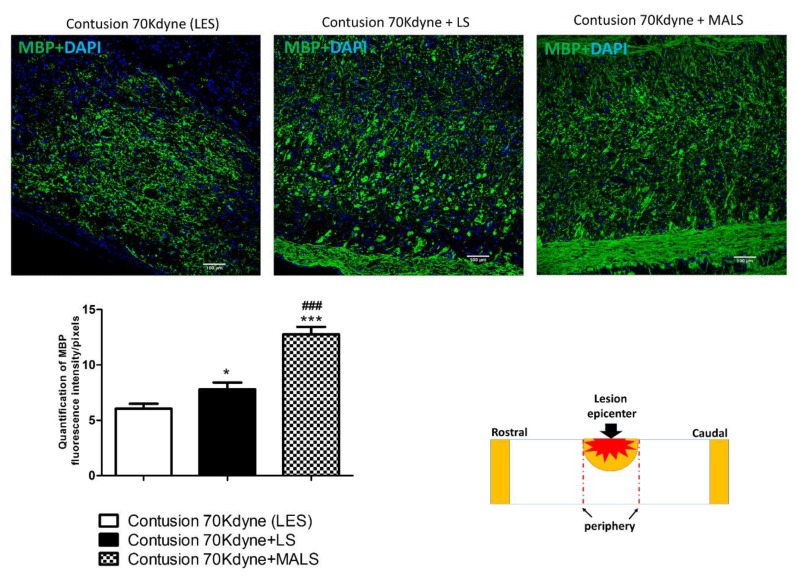
Protective action of MALS on myelin sparing in the injured cord. The image shows the protective action of MALS tissue in the epicenter of the injured cord (please see schematic representation). After animal perfusion, spinal cords were dissected, postfixed, and longitudinally sectioned by means of a cryostat. Spinal cord tissue sections were stained for myelin basic protein (MBP, green). The confocal microscope images for the spinal cord of lesioned animals and transplanted with LS or MALS tissue were obtained using the same intensity, pinhole, wavelength, and thickness of the acquisition. Scale bar: 100 µm. The graph reported shows the quantification of fluorescence with reference to MBP staining. Data is expressed as mean of twelve different fields (n = 4 mice; 3 fields/mouse for each condition). Values represent mean ± SD. We determined the statistical differences by means of one-way ANOVA test followed by Bonferroni’s post-test. *** *p* < 0.001; *p < 0.05 vs. LES; ### *p* < 0.001 vs. LS.

Myelin basic protein (MBP) is a fundamental factor present in the myelin-proteolipid shell of axons of oligodendrocytes and Schwann cells in the nervous system. In order to investigate the effect of MALS transplantation on white matter preservation, we stained spinal cord slices with the MBP antibody. We observed an increase in the MBP staining in LS and even more in the MALS treated mice, suggesting the counteraction of myelin loss and the maintenance of possible supportive elements for neuronal regeneration at the lesion epicenter (Figure 3). By using the staining for this marker, we evaluated the amount of myelin fibers, which appear to be increased significantly in LS treated mice with respect to controls, and even more so in MALS treated mice. Considered all together these evidences indicate cellular presence neighboring the lesion site, suggesting white matter sparing.

### 3.4. MALS Engraftment Promotes Both BrdU Incorporation and Neural Markers Immunoreactivity

**Figure 4:**
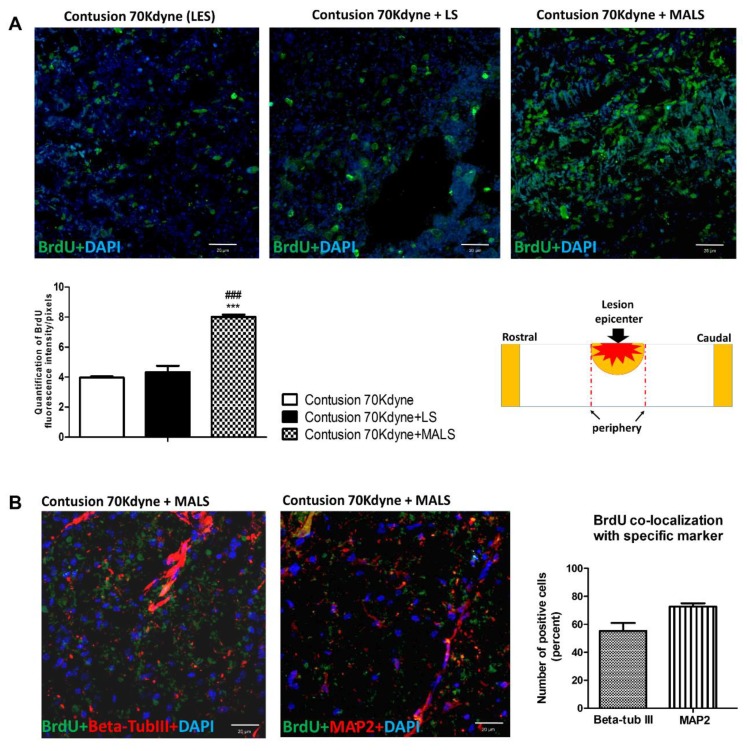
Transplanted MALS promotes an increase of BrdU labeled cells positive to neuronal markers. (**A**) The images show the BrdU positive cells (green) at the lesion epicenter (please see schematic representation) of contused not treated animals (LES), contused animals treated with lipoaspirate tissue, or contused animals treated with MALS tissue. Nuclei are stained with DAPI. The confocal microscope images for the spinal cord of the three groups were obtained using the same intensity, pinhole, wavelength, and thickness of the acquisition. Scale bar 20 µm. The graph reported shows the quantification of fluorescence with reference to BrdU staining. Data is expressed as mean of nine different fields (n = 3 mice; 3 fields/mouse for each condition). Values represent mean ± SD. We determined the statistical differences by means of one-way ANOVA test followed by Bonferroni’s post-test (*** *p* < 0.001 vs. LES; ### *p* < 0.001 vs. LS). (**B**) Spinal cord sections of MALS treated animals labelled with BrdU (green) were counterstained with anti-beta-tubIII or anti-Map2 antibodies (in red). The graph shows the percentage of BrdU-positive cells expressing the above neuronal markers. Data is expressed as mean of nine different fields (n = 3 mice; 3 fields/mouse). Values represent mean ± SD.

The above results raised an important question: did we with the MALS treatment create favorable enough conditions for the neuronal differentiation of endogenous stem cells? Ependymal stem cells are the only cells retaining stem cell potential in the adult spinal cord [45] and it appeared important to investigate their presence after SCI. Moreover, the same area of the cord should be enriched in neuronal precursors. To investigate the influence of fat transplantation on neurogenesis we evaluated the incorporation of BrdU at lesion site, which was administered 25 days after tissues engraftment. Animals were sacrificed 10 days later (day 35 after treatment) and BrdU incorporation was evaluated by immunodetection with monoclonal antibody in coronal spinal cord sections. The number of proliferating cells resulted increased in the MALS treated animals compared with LS or lesioned not treated animals (Figure 4A). This indicates that there is a higher number of proliferating cells in mice treated with MALS. Furthermore, we investigated the expression of neuronal markers beta-tubIII and MAP2 by BrdU labeled cells (Figure 4B). BrdU positive cells result highly positive to the expression of beta-tubIII and MAP2 markers (>70%), suggesting that MALS may promote the differentiation of newly generated cells towards also neuronal phenotype. 

**Figure 5:**
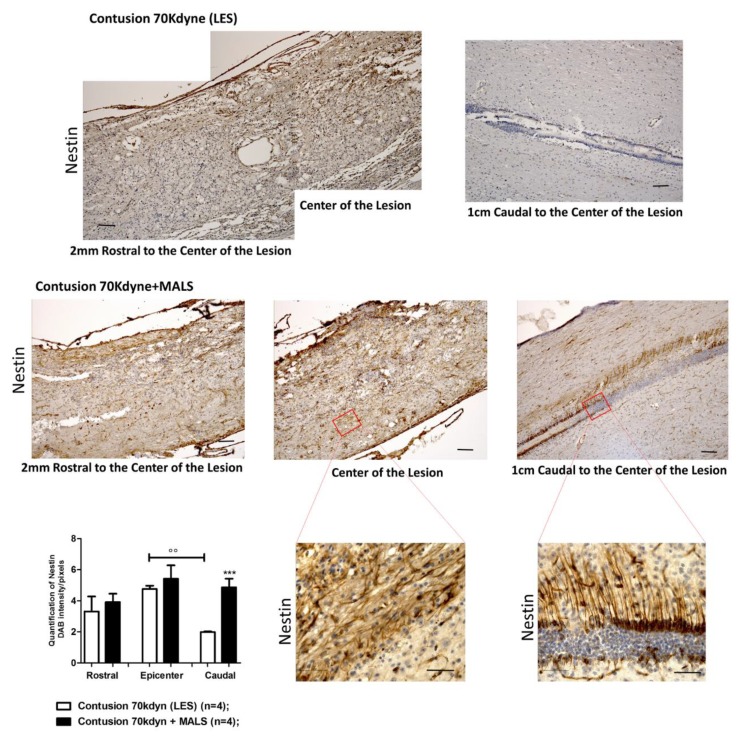
MALS engraftment leads to increased neurogenesis in the grafted cord. The image shows an increase in neural precursor cells at the lesion site of MALS transplanted animals, as demonstrated by the increase in Nestin positive cells. The graph reported shows the quantification of the distribution of Nestin’s expression along the grafted cord. Data is expressed as mean of twelve different fields (n = 4 mice; 3 fields/mouse). Values represent mean ± SD. We determined the statistical differences by means of ANOVA test followed by Bonferroni’s post-test. *** *p* < 0.001 vs. LES; °° *p* < 0.01 of LES caudal vs. LES epicenter. Symbols ($;°;*) indicate the corresponding area in Figure S2.

**Figure 6:**
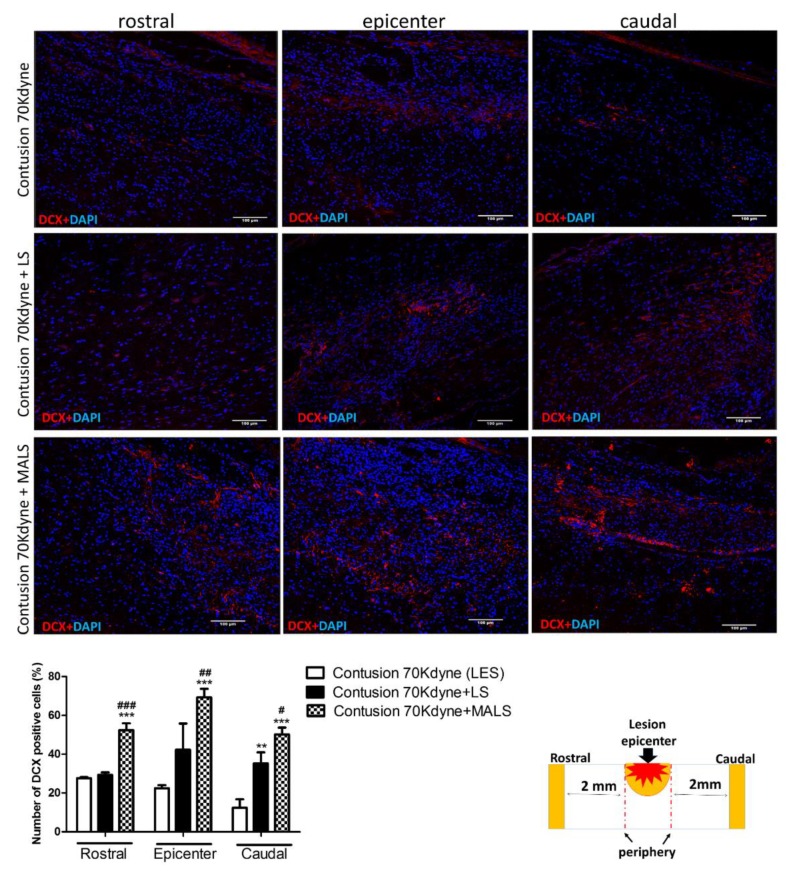
MALS tissue promotes re-growth of neurons. Spinal cord longitudinal sections were examined for DCX (red) expression 35 days after MALS transplant. Scale bar: 100 µm. The graph shows the quantification of positive cells (percentage) in sections taken at the lesion epicenter, 2 mm rostral or caudal to the lesion epicenter (please see schematic representation). Values represent mean ± SD. We determined the statistical differences by means of one-way ANOVA test followed by Bonferroni’s post-test. ** *p* < 0.01, *** *p* < 0.001 vs. LES, # *p* < 0.05, ## *p* < 0.01, ### *p* < 0.001 vs. LS. (n = 4 mice; 2 sections/mouse; 3 fields/section area).

Longitudinal spinal cord sections containing the ependymal canal were evaluated for the expression of nestin (Figure 5, Figure S2) and doublecortin (DCX) as marker of early neuronal differentiation (Figure 6). In the group of mice treated with MALS, all the investigated areas of the cords (the epicenter, 2 mm rostral and 1 cm caudal) resulted highly positive to nestin staining, and the marker resulted more abundantly expressed in MALS compared to LES animal group (Figure 5). The magnification of the ependymal canal, kept caudally to the lesion site (please see Figure S2), shows that more stem cells are stimulated to growth and regenerate the area after SCI (Figure 5; Figure S2). 

Doublecortin (DCX) is a microtubule associated protein expressed by neuronal precursors and immature neurons in embryonic and adult central nervous system, and neural precursors express DCX while actively dividing [46,47]. To further support the above evidence on MALS tissue’s protective effect on the degenerating spinal cord, cryostat sections of spinal cord of the three conditions (LES; LES+LS; LES+MALS), taken at 2 mm rostral and caudal to the lesion epicenter and in the center of the lesion, were examined for DCX expression 35 days after transplant (Figure 6). The immunoreactivity for DCX resulted increased in LS treated spinal cords and was noticeable at the lesion epicenter (Figure 6). MALS treated spinal cords resulted highly positive the DCX staining in the area of lesion epicenter and caudally, where it is especially concentrated in the ependymal canal. Spinal cords from lesioned not treated animals showed a very low positivity to the DCX staining (Figure 6). The quantification of DCX expression was performed and the graph shows a significant increase caudally to the lesion epicenter in spinal cords of MALS treated animals (Figure 6, Figure S3). 

**Figure 7:**
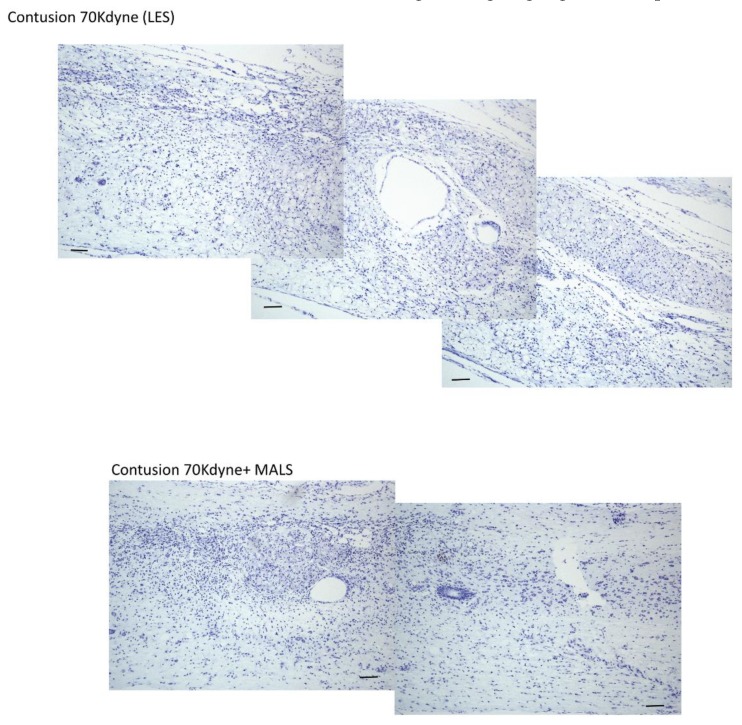
MALS transplanted spinal cords show increased neuronal presence cresyl violet staining of the lesioned spinal cord. The staining shows the presence of Nissl substance in the cytoplasm of neurons, more abundant at the epicenter of animals transplanted with MALS respect to LES. Scale bar: 100 µm. The picture is representative of labeling performed in two different sections (n = 4 mice; 2 sections/mouse for each condition).

The cresyl violet staining was applied to mark neuronal presence at the lesion site. Indeed, the staining highlights the presence of Nissl substance in the cytoplasm of neurons, and it is possible to appreciate how the staining is more evident in the epicenter of the animals transplanted with MALS respect to LES (Figure 7). This could suggest, after 35 days, an increase in the number of neurons present in animals treated with MALS, thus indicating an on-going regenerative process. 

**Figure 8:**
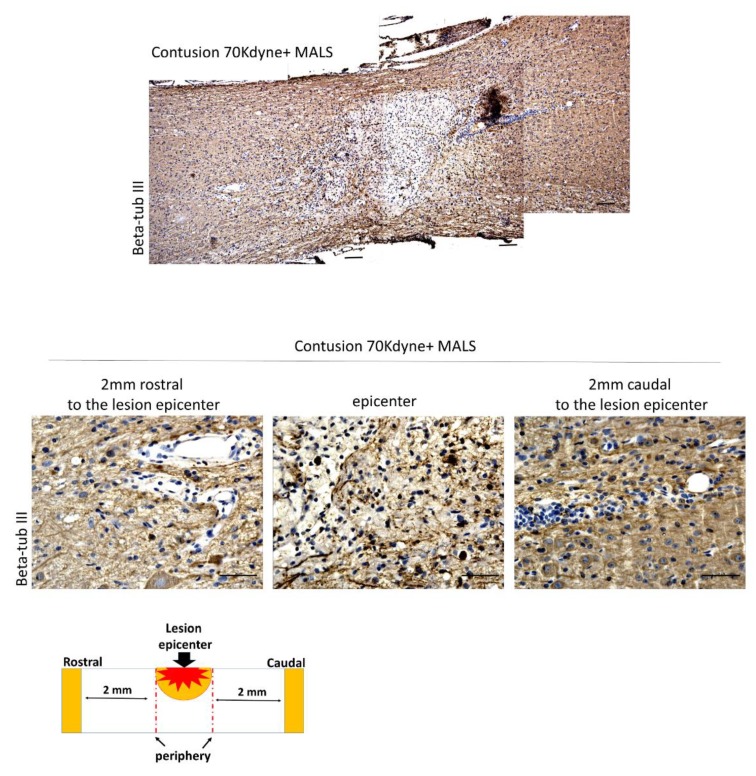
Spinal cords engrafted with MALS show neuronal presence at lesion site. The epicenter of the lesion and the surrounding areas (2 mm rostral and 2 mm caudal) were evaluated for the presence of neuronal markers expression beta-tub III. It is possible to appreciate beta-tub III positive cells in the analyzed regions. Scale bars: 100 and 50 µm. (n = 4 mice; 2 sections/mouse; 3 field/section).

Moreover, 35 days post injury the areas around the epicenter resulted positive to the staining for beta-tubulin III, a marker of neurons’ microtubules in the central nervous system (Figure 8). The positivity to beta-tubulin III further supports the fact that MALS in transplanted mice promoted the differentiation of neuronal progenitors into neurons.

### 3.5. Fluoro-Ruby Labeling Highlights Axonal Regeneration in the Injured Cord

**Figure 9:**
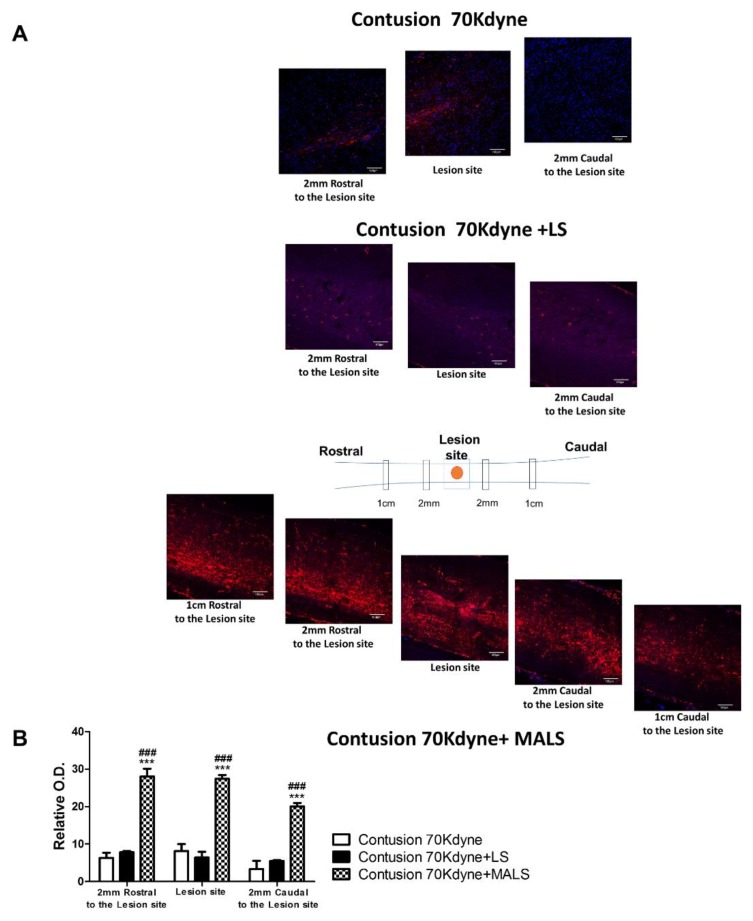
In vivo axonal transport recovery. (**A**) Qualitative images of anterograde axonal transport 35 days after contusion in lesioned non-treated animals (LES), or lesioned animals treated with LS and MALS. As described in Materials and Methods, Fluoro-Ruby was injected at T6/T7 25 days after lesioning and animal sacrificed 10 days later. Schematic reconstruction of spinal cord longitudinal sections of lesioned, lesioned + LS, and lesioned + MALS treated animals. Nuclei were stained with DAPI (blue). (**B**) The graph shows the quantification of fluorescence 2 mm rostral and caudal to the lesion and at the epicenter of the lesion. Quantification was performed in three animals per group 35 days after lesion. Statistical differences were determined by means of one-way ANOVA test followed by Bonferroni’s post-test. *** *p* < 0.001 vs. LES; ### *p* < 0.001 vs. LS.

To evaluate quantitatively anterograde axonal regeneration Fluoro-Ruby was injected in the rostral cord at T6/T7 level 25 days after injury and MALS or LS transplantation (see Section 2 for details; Figure 9) [48]. The animals were then sacrificed ten days after tracer injection. 35 days post injury, Fluoro-Ruby was picked up only by intact axons. Labelling occurred differently in the three conditions (Figure 9A). In lesioned animals, the tracer was picked up at the site of injection up to the lesion epicenter, confirming our previous observations [13]. In lesioned animals transplanted with LS, the uptake was present from the injection site to the epicenter of the lesion, and only slightly caudally to the lesion. On the contrary, in the group of animals treated with MALS the uptake was noticeable from the injection site to the caudal cord across the lesion epicenter (Figure 9A). In this case, it is possible to appreciate the highest number of labelled axons that cross the lesion epicenter. The quantification of Fluoro-Ruby labelled axons is reported in the graph and it shows a two-fold increase of axon labeling in the caudal cord proximal to the lesion (2 mm) of MALS-treated animals (Figure 9B). 

**Figure 10:**
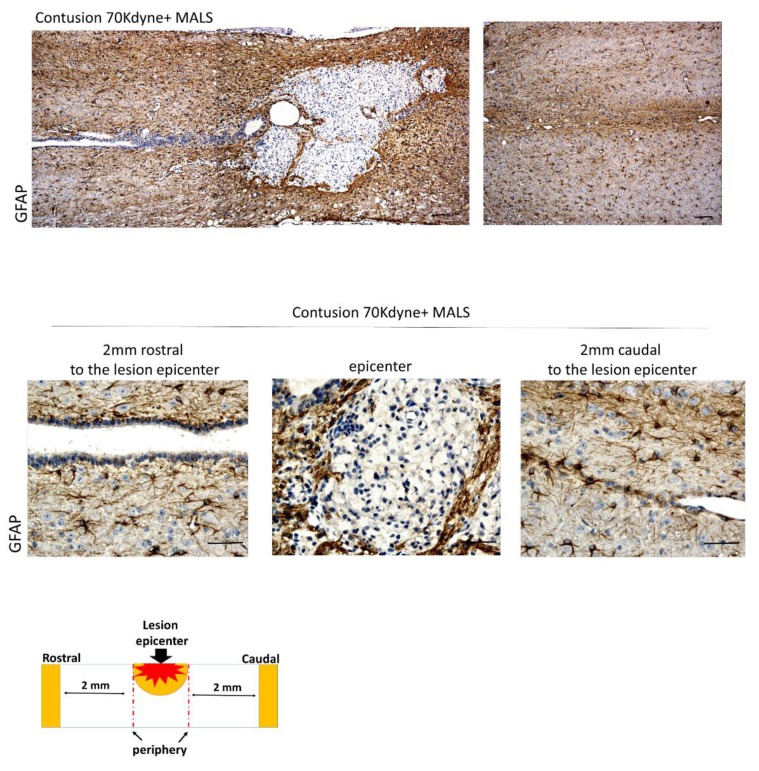
Glial scarring at the lesion site. The epicenter of the lesion and the surrounding areas (2 mm rostral and 2 mm caudal) were evaluated for the presence of GFAP. This indicates an ongoing scarring process. Scale bar: 100 and 50 µm. (n = 4 mice; 2 sections/mouse; 3 fields/section).

There is also a presence of GFAP positive cells, which has a role in repair after CNS injury, contributing to the formation of a glial scar at the lesion site. The presence of GFAP positive cells at the lesion site of MALS treated mice (Figure 10) indicates the presence of a physiological scarring process, aimed at repairing and taming the damage of the injury. 

### 3.6. MALS Engrafted Invades the Lesion Site in the Injured Spinal Cord

**Figure 11:**
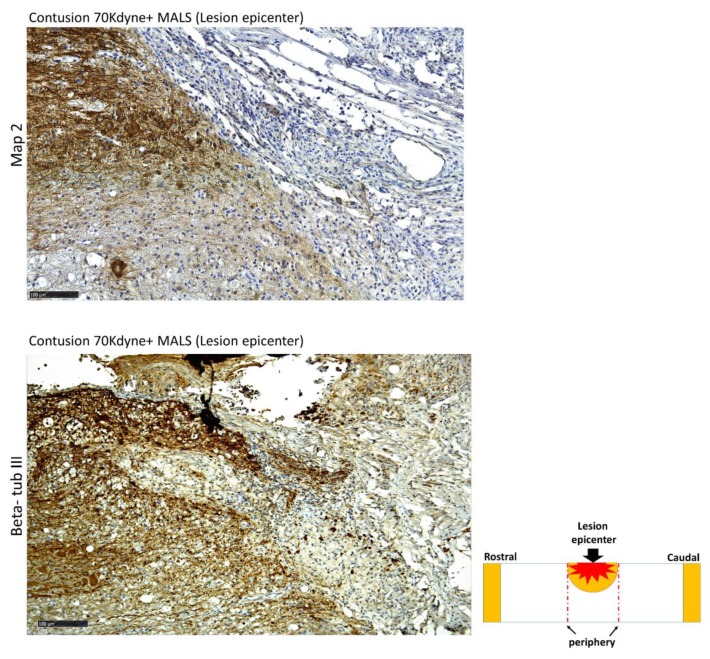
Synergistic interaction between MALS tissue and surrounding spinal cord microenvironment. The contact area between the lesion site and the MALS tissue shows positivity for two neuronal markers: Map2 and beta-tubulin III. Scale bar 100 µm. (n = 4 mice; 2 sections/mouse; 3 fields/section).

**Figure 12:**
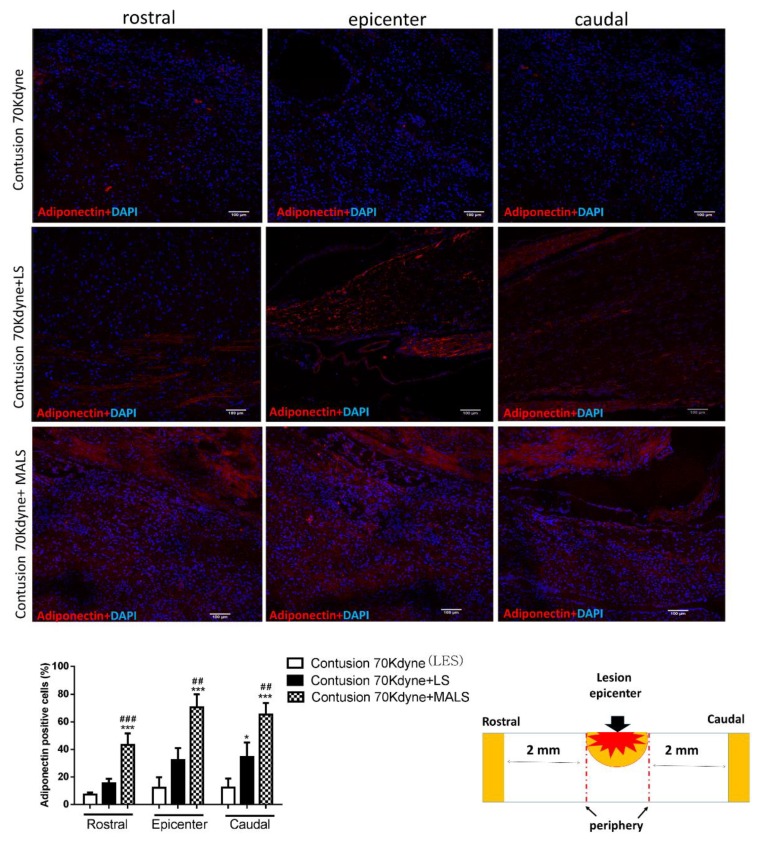
Engraftment of the LS and MALS tissue in the spinal cord-lesioned area. The images show the presence of adiponectin (red), a hormone typically produced by the adipose tissue. The staining was performed at the lesion epicenter and 2 mm caudal and rostral to the lesion site of animals transplanted with LS and MALS tissues. Scale bar: 100 µm. The graph reports the percentage of cells positive to the marker in the analyzed sections (please see schematic representation). Values represent the mean ± SD. We determined the statistical differences by means of ANOVA test followed by Bonferroni’s post-test * *p* < 0.05, *** *p* < 0.001 vs. LES; ## *p* < 0.01, ### *p* < 0.001 vs. LS.

**Figure 13:**
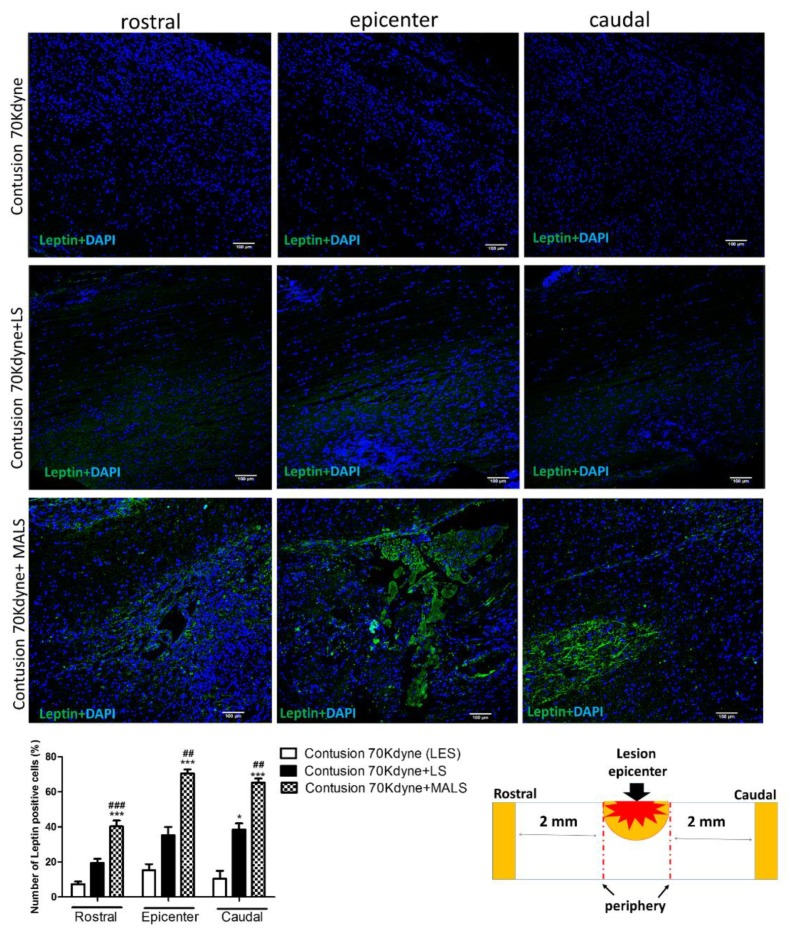
Engraftment of the MALS tissue in the spinal cord-lesioned area. The images show the presence of leptin (green), hormone typically produced by the adipose tissue. The staining was performed at the lesion epicenter and 2 mm caudal and rostral to the lesion site of animals transplanted with LS and MALS tissue. Scale bar: 100 µm. The graph reports the percentage of cells positive to the marker 2 mm rostral or caudal to the lesion epicenter (please see schematic representation). Values represent mean ± SD. We determined the statistical differences by means of ANOVA test followed by Bonferroni’s post-test. * *p* < 0.05, *** *p* < 0.001 vs. LES; ## *p* < 0.01, ### *p* < 0.001 vs. LS.

Thirty-five days after contusion, we investigated the engraftment capabilities of MALS and LS tissues on recipient spinal cords. Both LS and MALS were found to be vital and resulted engrafted at lesion site (Figure 11, Figure 12 and Figure 13). The MALS tissue seems to diffuse more efficiently through the injured spinal cord and a contact between the fat and the cord tissue is present. Figure 11 shows that the contact area between MALS and spinal cord is positive to two neural markers, Map 2 and beta-tubulin III, which are present also in the fat tissue, thus suggesting a cross-talk between the two tissues. 

The engraftment of LS and MALS tissues into the spinal cord-lesioned areas was investigated evaluating the presence of adiponectin and leptin. These hormones are mainly produced by the adipose tissue [49]. Figure 12 shows that adiponectin is present both in LS and MALS treated cords. In the case of LS engrafted cords, the staining is more limited to the lesion epicenter. Interestingly, in MALS engrafted cords, the distribution of the adiponectin staining is also appreciable 2 mm rostral and caudal to the lesion epicenter. The quantification reported in the graph shows the number of cells expressing adiponectin at the distribution of this marker is equal in this part of the injured spinal cord. 

Differently from adiponectin, leptin is expressed mainly rostral to the lesion epicenter and at the lesioned site (Figure 13). These evidences are confirmed by the quantification reported in the graph showing the number of cells positive to the marker. The quantification of cells positive to the two adipose marker was also confirmed by the quantification of the fluorescence intensity (Figure S4). All these results together indicate the engraftment of adipose tissue in the cord injured site, and a potential cross-talk between the two different tissues. 

## 4. Discussion

In this study, we aimed at evaluating a novel approach for spinal cord repair that employs mechanically activated adipose tissue transplanted at the lesion site. We observed that adipose tissue engraftments possess the physical properties to serve as a natural, three-dimensional space-filling material remaining localized at the injury site [26]. Differently from certain artificial scaffolds, adipose tissue fragments are not fragile, and their handling is, thus, convenient, with the implant being easily fitted into and above cavity [26]. The effects of transplanted mechanically activated adipose tissue (MALS) at the lesion site of animals suffering from acute traumatic SCI is directed to control inflammation acutely enhanced by traumatic lesioning, and its effects are comparable to those observed with EPO and corticosteroids [11,50]. The mechanical activation is obtained by orbital shaking at 97× *g* for 10 min, as we previously reported this procedure develops high anti-inflammatory properties in the fat with a decrease in pro-inflammatory agents’ expression [23]. Neuroinflammation is a physiologic response to SCI resulting in microglia activation, which secretes multi-functional immunoregulatory factors, most notably IL-1 alpha, IL-1 beta, and IL-6 families [13,15,51,52]. Neuroinflammation recalls into the cord inflammatory cells and increases permeability of the BBB [44,53,54,55]. The enhanced expression of anti-inflammatory cytokine TSG-6 exerts protective effects when administered in experimental diseases by reducing cytokine production and the invasion of infiltrating neutrophils and neutralizing CXCL8 [23,27]. Indeed, transplanted MALS downregulates alpha and beta IL-1, TNF-alpha, IL6, and IL8 mRNAs throughout the lesioned spinal cord. This corresponds also to the decrease of macrophages infiltration in the injury site. The transplanted MALS tissue survived in the lesion cord for a long time (35 days) in spite of the strongly unfavorable environment, that could have been modified by the taming of the inflammatory response and the release of anti-inflammatory molecules such as TSG6 [23]. These observations are in line with previous reports in which TSG6 produced by mesenchymal stem cells showed potent anti-inflammatory action in different conditions, such as lung damage [56] and mild traumatic brain injuries [57].

After SCI, there is a loss of myelin and oligodendrocytes, with the apoptosis of the latter that may proceed for several weeks [16,58,59,60,61,62]. Myelin regeneration, the process by which transplanted cells produce myelin around axons that have lost their myelin sheaths, has been targeted as a mechanism to enhance recovery following SCI. In the present work, we observed a small increase of myelin basic protein (MBP) positive cells in LS-treated mice, which was far more pronounced in MALS-treated mice. This situation was also very evident in the lesion epicenter suggesting a profound effect on white matter sparing favoring both axon survival and regeneration of lesioned axons. The positive effect of MALS transplantation on stem cells in injured spinal cord is confirmed by the activation of cell proliferation. It was also observed that the activated fat affected profoundly the fate of endogenous stem cells present in the ependymal canal; these cells differentiate and grow greatly with MALS treatment. This activation is shown by the increase in cells with nestin and doublecortin expression. Doublecortin (DCX) is a microtubule associated protein expressed by neuronal precursor cells and immature neurons in embryonic and adult central nervous system. Neural precursors express DCX while actively dividing [46,47]. The immunoreactivity for DCX was present in numerous cells 2 mm rostral to the lesion epicenter and in the epicenter itself, and is especially concentrated in the cells surrounding the ependymal canal. We might argue that MALS exerted simultaneously a possible neuroprotective action allowing the structural preservation, and neo-formation of a favorable milieu. This is suggested by the higher preservation of neuronal markers such as Beta-tub III and Map-2 at the site of interaction between MALS and spinal cord. 

The age-dependent decline in axon regeneration in the adult mammalian CNS is accompanied by increased immunoreactivity to glial fibrillary acidic protein (GFAP), a marker for reactive astrocytosis. In some cases, cell therapies following SCI cause a modification of astrogliosis with a reduction of GFAP immunoreactivity [13,15,63,64], yet it is unclear if this event is correlated to the promotion of axonal growth. GFAP-positive reactive astrocytes in proximity to SCI lesion sites were described as an unfavorable substrate for axon growth that could also secrete growth-inhibitory molecules [65]. On the other, it was noted that the prevention of astrocyte scar formation does not promote axonal regrowth across lesion [66], and may even cause a reduction of regenerative axons [17]. Indeed, reactive astrocytes help restrict secondary damage by forming a compact scar protecting outside tissue from a non-neural lesion core rich in inflammatory cells and fibroblasts [65,66,67]. MALS causes a reduction of diffused GFAP in the lesion cord, and such a reduction correspond to sites were activated fat has infiltrated into the cord as shown by leptin and adiponectin cellular staining. This also corresponds to the increased ependymal cells activation shown by nestin and DCX positivity. These cells appear to migrate from the intact cord to the lesion epicenter. In addition, in the grey matter there may be the neoformation of neurons deriving from the activated ependymal cells, which form new neural aggregates in the gray matter close to the lesion epicenter. 

In this study, we demonstrated for the first time that the treatment by the implant of heterologous adipose tissue with protective characteristics was possible and exerted a significant therapeutic effect when it was transplanted into mice affected by SCI. MSCs by definition must lack the expression of HLA II, which are responsible for the initiation of the immune response [68,69]. Moreover, the MSCs expression of soluble factor H, the complement regulatory proteins CD46, CD55, and CD59, allows MSC to be able to inhibit activation of the complement system to a certain extent [70,71]. This, together with the strong anti-inflammatory properties of MALS [23] avoided the inflammatory reaction which would otherwise be obtained in heterologous transplantation. The therapeutic effect was evaluated by employing the open field locomotion test attributing a score to the motor function recovery according to the Basso Mouse Scale (BMS) [37]. Our lesioning paradigm is well consolidated [13,15,34]. Our contusion procedure does not completely severe the cord, and in addition to the lateral and ventral pathways there are channels of survived spinal tissue, but the post-traumatic inflammation and hypoxia are favorable environment for secondary degeneration and render these portions a hostile milieu for axonal regrowth. It is, thus, conceivable that the prompt and rapid action of MALS renders these territories amenable to sustain neurogenesis as we report here. Betz and coworkers grafted adenovirus transduced fat to express BDNF and NT3 [26] reporting histological data suggesting the repair of damaged spinal cord injury. In our study, we report that the rapid attenuation of inflammatory process exerted by heterologous activated fat obtained by “minimal manipulation approach” [23] may be responsible of recovery of function. The previous work reported that genetically modified fat transplanted into injured spinal cord may counteract neuroinflammation [26]. Here we confirmed this suggested evidence by applying a more natural fat pad, and integrated the possible mechanism of action. Our data support the hypothesis that MALS favors neurogenesis. The distributions of both nestin and DCX staining through the spinal cord tissue near the lesion not only in the ependymal canal (neurogenic area) prompt us to suggest that MALS would functionally interact with the micro-environment of injured areas of the cord contributing to neurogenesis, not only in niches where neurogenesis occurs constitutively. This is supported also by the progressive morphological changes and by the expression of neuronal markers such as beta-tubulin III and MAP2 near to the fat pad. It could be also plausible that MALS allows the migration of meningeal cells (nestin and DCX positive cells) to the spinal cord parenchyma for the promotion of the repair process [72]. This interesting hypothesis will need further investigation in order to be demonstrated in this context.

The obtainment of MALS by a minimal manipulative approach has the positive characteristic of not modifying the original relevant characteristics of the tissue, especially the ones concerning its utility for reconstruction, repair and replacement surgery [73,74]. This tissue is not recognized as a medicinal product (drug), as there is only a minimal manipulation. Indeed, by definition, the international society of stem cell research (ISCCR) and the regulatory agencies (Food and Drug Administration and European Medicines Agency) consider cell therapy as a drug when there is more than a minimal manipulation of cells destined for application in a clinical setting or where the intended use of the cells is different to their normal function in the body [73,74,75]. Thanks to its obtainment with minimal manipulation, MALS can be highly useful in regenerative medicine. 

Together, these results demonstrate a strong potential for MALS in SCI, indicating a significant recovery of function likely due to a combined action of acute anti-inflammatory mechanisms and long-term stimulation of tissue regeneration, further studies are required to elucidate the mechanisms underlying their protective properties.

## Figures and Tables

**Table 1:** Sequences of oligonucleotides used for mRNAs expression evaluation.

Name	Sequence
m-IL1alpha F	ATGGCCAAAGTTCCTGACTTGTTTGAAGAC
m-IL1alpha R	GTTGCTTGACGTTCGTGATACTGTCACCCCG
m-IL1beta F	GGCAACTGTTCCTGAACTCAACTGTGAAAT
m-IL1beta R	CAGGTAGCTGCCACAGCTTCTCCACAGCCA
m-IL6 F	TCCAGTTGCCTTCTTGGGACTGATGCTGGT
m-IL6 R	AGTTTCAGATTGTTTTCTGCAAGTGCATCA
m-IL8 F	GCTCCTGCTGGCTGTCCTTAACCTAGGCAT
m-IL8 R	ATTGGGCCAACAGTAGCCTTCACCCATGGA
m-TNFalpha F	GACGTGGAACTGGCAGAAGAGGCACTCCCC
m-TNFalpha R	GAGGCCATTTGGGAACTTCTCATCCCTTTG
m-18S F	TTTTCGGAACTGAGGCCATG
m-18S R	TGGCAAATGCTTTCGCTCTG

## Supplementary Materials

The following are available online at https://www.mdpi.com/2073-4409/8/4/329/s1, Table S1: Prospective analysis (time) of functional recovery promoted by MALS. Figure S1: MALS engraftment counteracts macrophages invasion. Figure S2: neurogenesis shown in reconstructed picture of MALS grafted cord. Figure S3: quantification of fluorescence intensity for DCX immunoreactivity. Figure S4: quantification of fluorescence intensity for adiponection and leptin.
