# Supplementary material for: Neuroprotection, Recovery of Function and Endogenous Neurogenesis in Traumatic Spinal Cord Injury Following Transplantation of Activated Adipose Tissue
Source: Cells. 2019 Apr 8;8(4):329. doi: 10.3390/cells8040329 (PMC6523261; doi:10.3390/cells8040329)
Supplement: Supplementary file 1 [file cells-08-00329-s001.pdf]

Table S1. Prospective analysis (time) of functional recovery promoted by MALS. The groups were randomized, and the analysis was performed in double-blind fashion. Values represent mean ± SD (n = 10). Statistical differences were evaluated by means of two-way ANOVA test followed by Bonferroni's post-test.

**Days post MALS grafting**	**Behaviour (BMS scale)**	***P* value**
**1**	0.9 ± 0.18	
**3**	1.7 ± 0.20	$p < 0.001$vs. day 1
**7**	2.9 ± 0.29	$p < 0.001$vs. day 1<br>$p < 0.001$vs. day 3
**14**	4.1 ± 0.46	$p < 0.001$vs. day 1<br>$p < 0.001$vs. day 3<br>$p < 0.001$ vs. day 7
**18**	4.7 ± 0.52	$p < 0.001$vs. day 1<br>$p < 0.001$vs. day 3<br>$p < 0.001$ vs. day 7<br>$p < 0.05$ vs. day 14
**21**	4.9 ± 0.53	$p < 0.001$vs. day 1<br>$p < 0.001$vs. day 3<br>$p < 0.001$ vs. day 7<br>$p < 0.001$ vs. day 14<br>*p* = n.s. vs. day 18
**28**	5.4 ± 0.38	$p < 0.001$vs. day 1<br>$p < 0.001$vs. day 3<br>$p < 0.001$ vs. day 7<br>$p < 0.001$ vs. day 14<br>$p < 0.01$ vs. day 18<br>*p* = n.s. vs. day 21

**35**	5.6 ± 0.37	$p < 0.001$vs. day 1<br>$p < 0.001$vs. day 3<br>$p < 0.001$ vs. day 7<br>$p < 0.001$ vs. day 14<br>$p < 0.001$ vs. day 18<br>$p < 0.01$ vs. day 21<br>p = n.s. vs. day 28

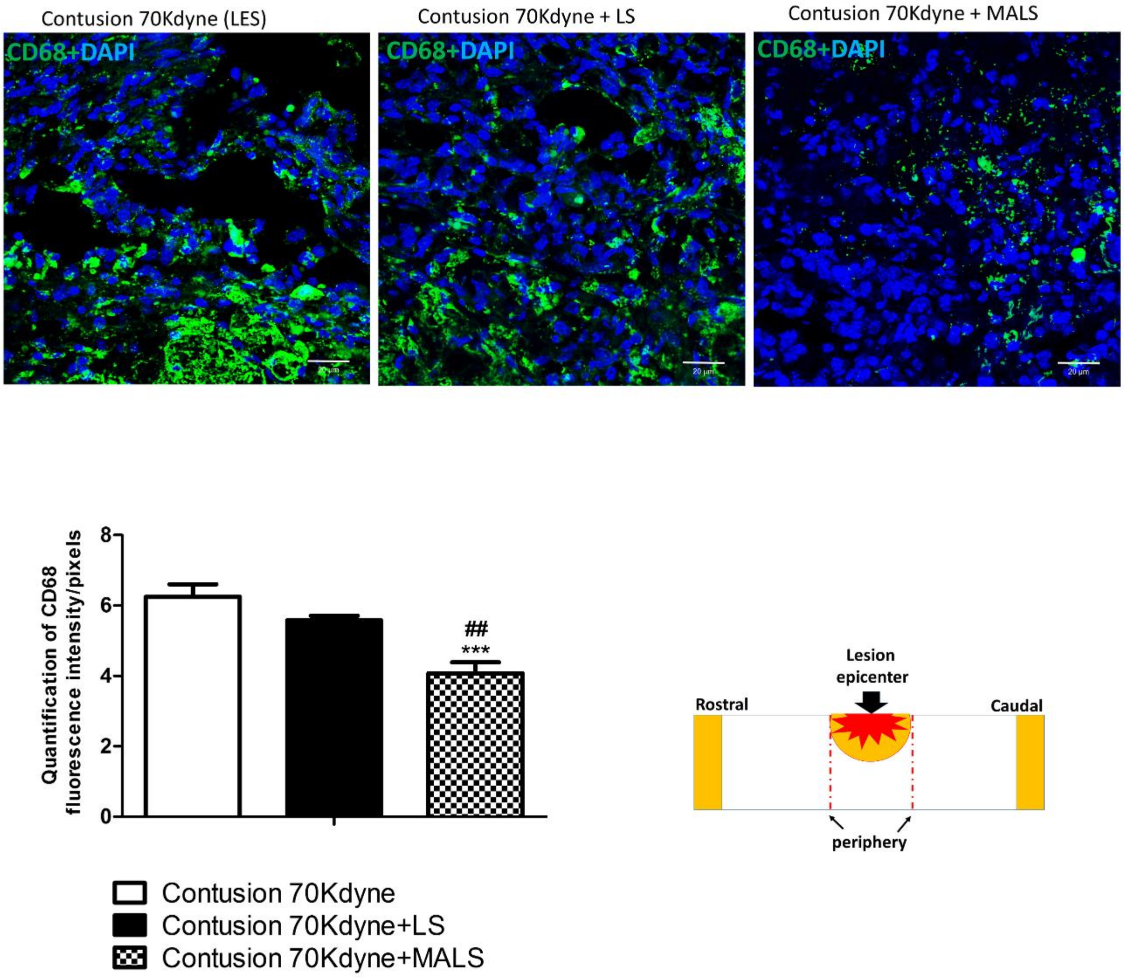

Figure S1. MALS engraftment counteracts macrophages invasion. Immunohistochemistry analysis of coronal sections obtained from the spinal cords of contused non-treated animals, (LES), contused animals transplanted with LS or MALS. The cord sections were labeled with the antibody CD68. Quantification was done by ImageJ (NIH. Bethesda, MD, USA) picture analysis software and reports the fluorescence intensity/pixel expressed by the studied markers (n = 3 mice each group; three fields for mouse). Values represent mean ± SD. Statistical differences were determined by means of ANOVA test followed by Bonferroni's post-test. *** $p < 0.001$ vs. LES; ## $p < 0.01$ vs. LS.

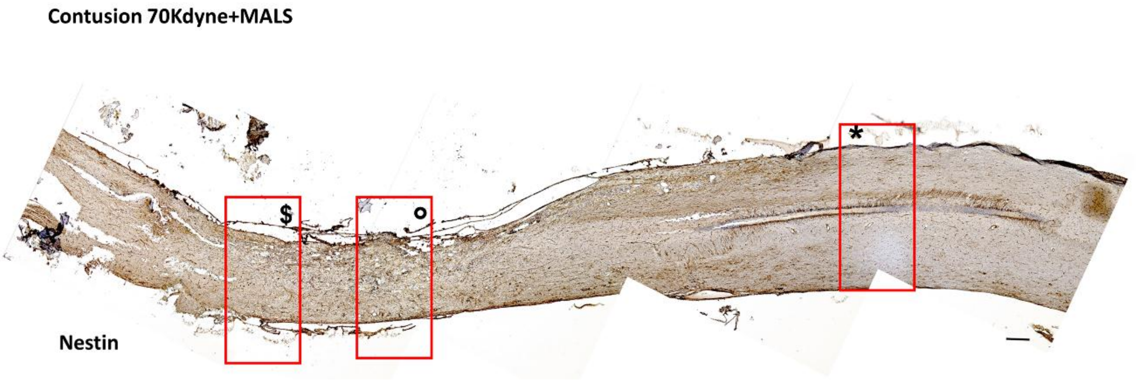

Figure S2. Neurogenesis shown in reconstructed picture of MALS grafted cord. The figure shows an increase in neural precursor cells in three different areas, the epicenter, 2 mm rostral and 1 cm caudal areas to the lesion, of MALS transplanted animals. The neurogenesis is demonstrated by the increase in nestin-positive cells in the areas surrounding the graft. Scale bar 100 μm. (n = 4 mice; 2 sections/mouse; 3 fields/section area). Symbols ($; °; *) refer to the magnification reported in Figure 5.

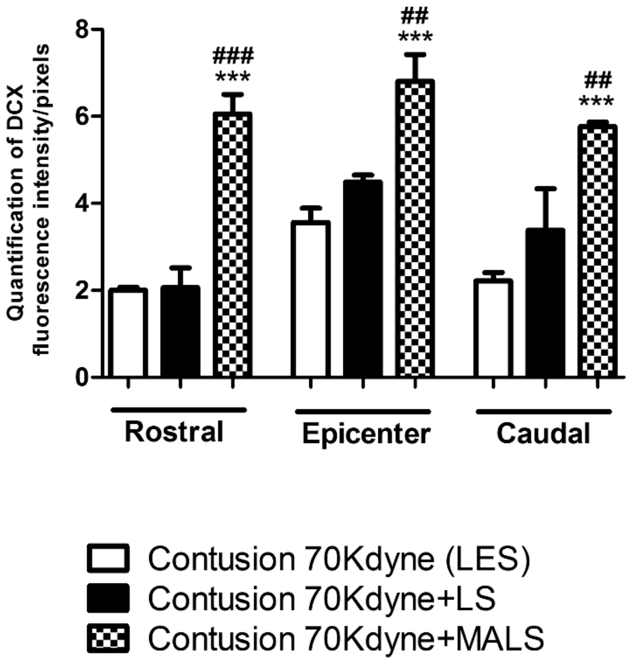

Figure S3. Quantification of fluorescence intensity for DCX immunoreactivity. The quantification of immunoreactivity was performed in sections taken at the lesion epicenter, 2 mm rostral or caudal to the lesion epicenter (please see schematic representation in Figure 6). Values represent mean ± SD. We determined the statistical differences by means of ANOVA test followed by Bonferroni's post-test. *** $p < 0.001$ vs. LES; ## $p < 0.01$, ### $p < 0.001$ vs. LS. (n = 4 mice; 2 sections/mouse; 3 fields/section area).

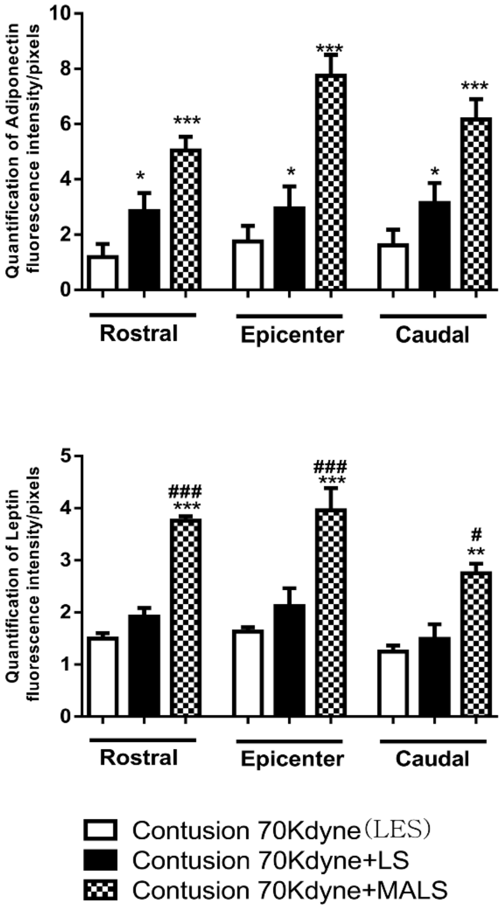

Figure S4. Quantification of fluorescence intensity for adiponection and leptin. The immunoreactivity of adiponectin is reported in the upper panel and that of leptin in the lower panel. The quantification of immunoreactivity was performed in sections taken at the lesion epicenter, 2 mm rostral or caudal to the lesion epicenter (please see schematic representation in Figures 12 and 13). Values represent mean ± SD. We determined the statistical differences by means of ANOVA test followed by Bonferroni's post-test * $p < 0.05$, ** $p < 0.01$, *** $p < 0.001$ vs. LES; # $p < 0.05$, ### $p < 0.001$ vs. LS.
